# Control and target gene selection for studies on UV-induced genotoxicity in whales

**DOI:** 10.1186/1756-0500-6-264

**Published:** 2013-07-09

**Authors:** Laura M Martinez-Levasseur, Diane Gendron, Robert J Knell, Karina Acevedo-Whitehouse

**Affiliations:** 1 Institute of Zoology, Regent’s Park, London NW1 4RY, UK; 2 School of Biological and Chemical Sciences, Queen Mary, University of London, Mile End Road, London E1 4NS, UK; 3 Current Address: Department of Biology, Trent University, 1600 West Bank Dr, Peterborough K9J 7B8, Canada; 4 Centro Interdisciplinario de Ciencias Marinas, Instituto Politécnico Nacional, Av. IPN s/n, Playa Palo de Sta Rita, La Paz, BCS 23000, México; 5 Unit for Basic and Applied Microbiology. School of Natural Sciences, Autonomous University of Queretaro, Queretaro 76230, México

**Keywords:** Gene expression, qPCR normalization, Internal control genes, HSP70, P53, KIN, Solar ultraviolet radiation, Whales, Skin biopsy

## Abstract

**Background:**

Despite international success in reducing ozone-depleting emissions, ultraviolet radiation (UV) is not expected to decrease for several decades. Thus, it is pressing to implement tools that allow investigating the capacity of wildlife to respond to excessive UV, particularly species like cetaceans that lack anatomical or physiological protection. One approach is to examine epidermal expression of key genes involved in genotoxic stress response pathways. However, quantitation of mRNA transcripts requires previous standardization, with accurate selection of control and target genes. The latter is particularly important when working with environmental stressors such as UV that can activate numerous genes.

**Results:**

Using 20 epidermal biopsies from blue, fin and sperm whale, we found that the genes encoding the ribosomal proteins L4 and S18 (*RPL4* and *RPS18*) were the most suitable to use as controls, followed by the genes encoding phosphoglycerate kinase 1 (*PGK1*) and succinate dehydrogenase complex subunit A (*SDHA*). A careful analysis of the transcription pathways known to be activated by UV-exposure in humans and mice led us to select as target genes those encoding for *i*) heat shock protein 70 (*HSP70*) an indicator of general cell stress, *ii*) tumour suppressor protein P53 (*P53*), a transcription factor activated by UV and other cell stressors, and *iii*) KIN17 (*KIN*), a cell cycle protein known to be up-regulated following UV exposure. These genes were successfully amplified in the three species and quantitation of their mRNA transcripts was standardised using *RPL4* and *RPS18*. Using a larger sample set of 60 whale skin biopsies, we found that the target gene with highest expression was *HSP70* and that its levels of transcription were correlated with those of *KIN* and *P53*. Expression of *HSP70* and *P53* were both related to microscopic sunburn lesions recorded in the whales’ skin.

**Conclusion:**

This article presents groundwork data essential for future qPCR-based studies on the capacity of wildlife to resolve or limit UV-induced damage. The proposed target genes are *HSP70*, *P53* and *KIN*, known to be involved in genotoxic stress pathways, and whose expression patterns can be accurately assessed by using two stable control genes, *RPL4* and *RPS18*.

## Background

A current and significant threat to marine ecosystems across the globe is the high level of solar ultraviolet radiation (UV) that continues to reach our biosphere [1], a situation that is not expected to change for several decades [2,3]. Recently we showed that cetaceans develop macro and microscopic skin lesions as a consequence of solar exposure [4], a study that widened the known range of marine species that can be affected by UV exposure [1,5]. Taking into consideration that many cetacean species are listed as endangered or threatened by the International Union for the Conservation of Nature, it is pressing to implement tools that allow us to evaluate their capacity to respond to current stressors [6], including excessive UV. One potential avenue for such studies is to examine the expression of key genes involved in genotoxic stress response pathways.

Continuous and unresolved exposure to UV damages DNA, in turn activating a network of interactive pathways that include participation of aberrant DNA sensors, signal transducers and effectors, which interact to execute appropriate responses [7]. Although the identities of the sensors are still unclear, transducers include four sets of conserved proteins, namely phospho-inositide kinases, check point kinases 1 and 2 and the group of BRCT (breast cancer C-terminal repeats) proteins [7]. Effectors involved in DNA repair, transcription regulation and cell cycle control, comprise proteins such as the tumour suppressor protein P53 [7,8]. This interactive network that involves hundreds of genes is complex [7,9] and to our knowledge has not been studied in wild mammals.

Real-time or quantitative PCR (hereafter qPCR) is currently the accepted method for quantifying mRNA transcripts [10]. However, despite the technique’s accuracy, sensitivity and speed [11], variations in the amount and integrity of starting material, transcription- and amplification efficiency rates, as well as the occurrence of inhibitors, can lead to quantitation errors. In this context, normalization of data is an essential step that must precede gene expression quantitation [10-12]. Most studies use endogenous reference genes as an internal control to calculate relative expression values of the genes of interest. Such internal controls should not vary in their expression levels amongst individuals, particularly under experimental conditions relevant to the question of interest. Accurate selection of reference genes is therefore central to interpreting quantitative PCR results.

A recent study on free-ranging striped dolphins tested the expression stability of ten commonly used control genes in skin biopsies and found that the genes coding for the tyrosine 3-monoxygenase (*YWHAZ*) and glyceraldehyde-3P-dehydrogenase (*GAPDH*) were the most reliable ones followed by those coding for the ribosomal proteins S18 (*RSP18*) and the ribosomal proteins L4 (*RPL4*) [13]. However, a second study that evaluated gene expression changes associated with organochlorine exposure in striped dolphin fibroblast cultures showed that the most reliable gene was the one encoding succinate dehydrogenase complex subunit A (*SDHA*) [14]. These studies illustrate how, depending on the purpose of the study and the target tissue to be analysed, different genes might be better suited as controls. Here, as part of a larger ongoing study on the effects of exposure to UV on whales [4], we examined expression levels and assessed stability of selected reference genes in skin biopsies collected from three species of large whales and used two of the most suitable genes to study expression patterns of key genes involved in genotoxic stress pathways.

## Methods

A schematic representation of the general method used in this article is provided in the Additional file 1: Figure S1.

### Skin biopsy sampling

Skin biopsies were collected from blue, fin and sperm whales in the Gulf of California, Mexico, between January and June of 2007 to 2009. Samples were collected using a 7 mm stainless-steel dart and a crossbow. An epidermal sub-sample was immediately stabilised in RNA later (Qiagen, UK) and ultrafrozen until processing. This study is registered as project WLE/0405 at the Institute of Zoology and conforms to the regulations on Animal Ethics. Samples were collected under permits SGPA/DGVS/00506/08, SGPA/DGVS/09760/08 and SGPA/DGVS/08021/06 issued by the Mexican Secretaría del Medio Ambiente y Recursos Naturales (SEMARNAT).

### RNA extraction and cDNA transformation

Total RNA was extracted using the RNeasy® Mini Kit (Qiagen, UK) according to the manufacturer’s instructions. The quantity of RNA obtained was determined for each sample by measuring optical density (OD) with a Nanodrop® ND-1000 UV–vis spectrophotometer (Thermo Scientific, UK). OD 260/280 and 260/230 ratios were used to evaluate RNA purity. Presence of intact RNA subunits 28S and 18S were checked by automated capillary-electrophoresis, using QIAxcel (Qiagen,UK; see Additional file 1: Figure S2). Before performing reverse transcription, all samples were diluted to a final concentration of 50 ng/μl as done previously [15]. Complementary DNA (cDNA) was obtained by reverse transcription using the QuantiTect® Reverse Transcription Kit (Qiagen, UK). This procedure includes a first step of DNA digestion. Only one retro-transcription was run per sample. Prior to use, cDNA was diluted 1:25 with nuclease free water and conserved at −20°C.

Twenty individual RNA samples (belonging to 7 blue whales, 7 fin whales and 6 sperm whales) were selected to measure expression of internal control genes. A total of 60 samples (belonging to 22 blue whales, 22 fin whales and 16 sperm whales) that included the 20 samples selected for the previously described analysis, were used to measure expression of target genes. The maximum and minimum RNA concentrations obtained for the samples selected were 634 ng/μl and 51 ng/μl (mean for all samples: 233 ng/μl ± 42.38 SE). The samples showed an absorbance ratio at 260/280 nm between 2.1 and 1.81, and an absorbance ratio at 260/230 nm greater than 0.95 except for four samples with an absorbance ratio of 0.85, 0.75, 0.74 and 0.59. Criteria for inclusion of the sample in the study include the presence of one or two intact bands during electrophoresis (see Additional file 1: Figure S2).

### Internal control gene candidates

We identified potentially-suitable internal control genes as those whose levels of expression are known to not be involved in any process related to UV exposure and that have been shown to be “stable” in other marine mammal species. The primer sets of the four control genes that fitted these criteria were obtained from a previous study conducted on striped dolphins [13], being *RSP18*, *RPL4*, *SDHA* and *PGK1* (phosphoglycerate kinase 1). Although *GAPDH* and *YWHAZ* have been reported as reliable control genes in dolphins [13] and were used recently in a study that assessed toxicological stress in fin whales [16], these genes were not included in our study because their expression has been shown to be affected directly or indirectly by UV exposure [17-19]. In vitro and in vivo studies that have examined human keratinocyte responses to solar irradiation showed that levels of *GAPDH* expression shifted significantly 24 h post irradiation [19,20]. Another study demonstrated low stability of *GAPDH* in UV irradiated keratinocytes [21], and, perhaps more importantly, the heat shock protein 70, an indicator of cellular stress [22], is known to alter the cellular amount of *GAPDH*[23]. Finally, *YWHAZ* not only interacts with the process of apoptosis [24] but has also been proved to be involved with the skin carcinogenesis process [17]. Instead, we selected *RSP18* and *RPL4*, the next most stable genes in dolphin skin [13]. Furthermore, although *PGK1* was previously considered less suitable as a control gene in striped dolphin skin [13], we selected it for our study because, together with *SDHA*, it is considered the most reliable control gene when studying the effects of exposure to UV-B radiation on human keratinocytes [21]. The selected primer pairs were commercially synthesized and tested for specificity in the three whale species as described below.

### Target gene candidates

Important genes involved in the complex UV-response pathway are those encoding the heat shock proteins (HSPs), also called stress proteins. HSPs are involved in the recovery of proteins that can unfold under stress [22,25]. HSPs can either repair the damaged proteins by refolding their structure, or can degrade them if damage is too extensive. The HSPs are also involved in intracellular protein transport between compartments and disposal of old proteins as well as in generating an immune response as they participate in the presentation of abnormal peptides (i.e. antigens) to immune effectors on the surface of abnormal cells [26]. The different families of HSPs, classified according to their structure, function and weight (in kilodaltons), include HSP100, HSP90, HSP70, HSP60, HSP40 and the small heat shock proteins family. One of the most studied HSPs is HSP70, which is the major stress-induced member of the family, specifically involved in protein-folding and protein membrane transport [22]. Humans and laboratory animals studies have showed that under severe UV irradiance, the gene coding for HSP70 is over-expressed and helps to protect against UV-induced epidermal damage, including apoptosis and DNA damage [27].

To minimize the number of heritable mutations transferred from one cell to its daughters, the structure of chromosomes is continuously under surveillance. When damage is detected, repair and cell-cycle progression are coordinated [7]. Protein P53, also termed ‘tumour suppressor protein’ because mutations of this gene can promote cancer [8,28], is actively involved in different response pathways including cell cycle arrest, DNA repair and, when unrepairable, apoptosis [8]. P53 is a central transcription factor in cellular stress responses and its synthesis is controlled by dozens of other proteins [29]. One of P53’s most important transcriptional targets is the cyclin-dependent kinase inhibitor p21, which can provoke cell-cycle arrest at G1 phase [29]. P53 also participates, via transcriptional regulation and direct interaction, in DNA repair mechanisms such as nucleotide excision repair (NER), although it has been shown that P53 is not always essential to NER [29]. P53 also induces the expression of *DDB2* and *XPC* genes, which encode factors of the global genome repair mechanism (GGR) [9,29]. While programmed cell death can occur independently of P53, this protein is involved, via various routes, with apoptosis, its most important suppressive function [8,29]. Finally, P53 is also involved in the tanning response [30].

A gene recently found to be implicated in cellular responses to UV-induced damage is the gene coding for KIN17 protein (hereafter *KIN*). The *KIN* gene is expressed in all tissues and its expression significantly increases after UV exposure [31,32]. Experimental trials have shown that DNA-bound KIN protein accumulates 24 h after irradiation and that KIN can arrest the cell cycle prior to DNA replication [33,34]. It has been proposed that the KIN protein helps to overcome the perturbation of DNA replication in unrepaired DNA sites [33].

### Target gene primer design

Primers were designed for the three selected target genes. For each gene, cDNA sequences listed for other species were searched for in the NCBI GenBank database (http://www.ncbi.nlm.nih.gov). For *KIN*, primers were designed by aligning highly conserved exonic regions of this gene in cow, horse, chimpanzee, mouse and human genomes (see Additional file 1). For *P53*, primers were designed by aligning dolphin, cow, pig and human sequences (see Additional file 1). For *HSP70* primers, we used the cDNA sequence reported for a north Atlantic Right whale [GenBank: ES556841.1] [35] (see Additional file 1). Sequences were aligned using the free Multiple Alignment software ClustalW (http://www.ebi.ac.uk/Tools/msa/clustalw2/). Primer pairs were designed within conserved regions (see Additional file 1), ideally spanning two exons to avoid DNA amplification. The primers were targeted to amplify 100–200 nucleotides in order to reduce potential noise caused by eventual RNA degradation and to decrease variation during qPCR. Each primer was 18–24 bp length, had 50–55% GC composition, had a melting temperature of 60°C and ended with a G or C base (3’). Occurrence of hairpins, homodimers and heterodimers were checked in the Integrated DNA technology freeware (IDT, http://eu.idtdna.com/analyzer/Applications/OligoAnalyzer). The primers successfully amplified in the three whale species and generated a single and well-defined band and a unique qPCR dissociation curve. Specificity of the primers was further confirmed by bi-directional sequencing of amplified products.

### PCR validation

For each species, each primer pair was tested in two randomly selected samples by independent PCRs. Volume per reaction was 12.5 μl and contained 1× PCR buffer (Tris-Cl, KCl, (NH_4_)_2_SO_4_ and MgCl_2_; Qiagen, UK), 0.2 mM dNTPs (Bioline, UK), 0.4 μM of each primer, 0.325 U of HotStarTaq®Plus DNA polymerase (Qiagen, UK) and 1 μl of cDNA. The PCR conditions were 95°C for 5 min, 35 cycles of 94°C for 1 min, 60°C for 45 s, 72°C for 30 s, and a final extension at 72°C for 10 min. Amplification products were run on a 2% agarose gel stained with 2.5× SYBR® Safe DNA stain gel (corresponding to 0.1 μl per ml; Invitrogen, USA). Fragments were excised and cleaned using the QIAquick® gel extraction kit (Qiagen, UK) before being sent for bi-directional Sanger sequencing (Cogenics, UK). Each sequence obtained (see Additional file 1) was analysed in the Basic Local Alignment Tool (BLAST, http://www.ncbi.nlm.nih.gov/BLAST) and it was confirmed that the PCR product amplified corresponded to the gene targeted.

### Standard curve and amplification efficiency

We used PCR products as a template for the construction of the standard curves for each of the genes tested. For this, three amplified products of each gene were run on a 2% agarose gel and the excised bands cleaned using the QIAquick® gel extraction kit (Qiagen, UK). PCR quantity was measured with the Nanodrop® ND-1000 UV–vis spectrophotometer (Thermo Scientific, UK) and dilutions were made to obtain stocks containing 10^2^ to 10^8^ copies of PCR product per μl. Each dilution was run in triplicate in a 7300 Real-Time PCR System (Applied Biosystems, UK) as described below. The logarithm of the product quantity obtained for each threshold value (Ct) was plotted against the Ct values to obtain the linear correlation coefficient (R^2^) for each gene. The slope of the curve was used to calculate qPCR amplification efficiencies (E=10^1/-slope^-1) for each set of primers [36].

### Real-time quantitative PCR (qPCR) using SYBR green

Real-time quantitative PCR (qPCR) was performed in a 7300 Real-Time PCR System (Applied Biosystems, UK) using Power SYBR Green PCR Master Mix (Applied Biosystems, UK). The total volume of each qPCR reaction was 10 μl, which included forward and reverse primers (500 nM), 1× Power SYBR Green PCR Master Mix (Applied Biosystems, UK) and the cDNA sample (2 μl of a 1:25 cDNA dilution). For each control gene, a 96-well reaction plate was set up to include all 20 samples, seven serial dilution points (10-fold step) of the same gene and three no-template controls (NTC) to ensure detection of inadvertent contamination. For the target gene analyses, each plate contains all the target genes and control genes set up for four samples and three no-template controls (NTC). Three RT-negative controls were run in the first plate to confirm that DNA elimination was successful. All samples were run in triplicate.

Cycling conditions were an initial 2 min at 50°C, followed by 15 min at 95°C, and 40 cycles of 15 s at 95°C, 1 min at 60°C and 1 min at 72°C. A melting curve analysis (95°C/15 sec; 60°C/1 min; 95°C/15 sec; 60°C/15 sec) was added at the end of the final cycle to detect non specific amplifications (see Additional file 1: Figure S3). Threshold values (Ct) and their transformation to quantities (Qt) were automatically determined with the 7300 Real-Time PCR System software (Applied Biosystems, UK). The mean of the triplicate reactions were calculated for each sample (standard deviation = ± 10% of the mean).

### Analysis of internal control gene expression stability

Gene expression values were analyzed using the freeware packages BestKeeper (http://gene-quantification.com/bestkeeper.html), geNorm (http://medgen.ugent.be/~jvdesomp/genorm) and NormFinder (http://www.mdl.dk/publicationsnormfinder.htm). The algorithms used by these packages have been developed for a minimum of three genes [12,37,38].

Briefly, BestKeeper ranks the control gene candidates according to the standard deviation of their Ct-value (SD_Ct value_). The correlation (Pearson correlation coefficient and probability) between each gene and index, corresponding to the geometric mean of the Ct-value of all suitable candidate genes, were calculated in order to determine the best suited genes [37]. For this, BestKeeper assumes that the Ct-value for each gene is normally distributed, an assumption that was confirmed by a Shapiro test using the R software [39]. The software geNorm ranks the candidate genes according to their average expression stability M. Briefly, a variation parameter V_*jk*_ is calculated for every combination of two internal control genes *j* and *k*. V_jk_ is equal to the standard deviation (SD) of the sum of the logarithmic transformed level expression ratio of the two tested genes measured for each sample *i* (see Equation 1) [12].

(1) $$\left(\forall j,k\in \left[1,n\right]\;\mathrm{and}\;j\neq k\right)\;:V_{\mathrm{jk}}=\mathrm{SD}\left[\log_{2}{\left(\frac{a_{\mathrm{ij}}}{a_{\mathrm{ik}}}\right)}_{i=1\to m}\right]$$

M_*j*_ is determined for each gene *j* as the arithmetic mean of all V_*jk*_[12]. Ideally, the expression ratio of two tested genes is identical in all samples. Increasing variation in ratio corresponds to decreasing expression stability [12]. Finally, normFinder estimates the overall expression variation of the control gene candidates and rank those according to their expression stability [38].

For geNorm and NormFinder, we used transformed Ct values corresponding to the quantities obtained with the standard curve [12,38], whereas raw Ct values were used for BestKeeper [37].

### Analysis of target gene expression

Gene expression levels were analysed using the relative quantification method (level of expression of the target gene relative to internal control genes) that is based on the ΔCt method (Ct _target gene_- geometric mean Ct_control genes_) [12,36]. In order to control for inter-plate qPCR variations, which might reflect unintentional grouping of the data and potentially hide effects on the levels of gene expression, we used linear mixed effect modelling [40] using the *lme* function in the *nlme* package [41] and defined ‘plate’ as a random factor. Models were built in R [39] and we used a top-down strategy to determine which variables explained a significant fraction of the data [40]. Violation of normality and homoscedasticity assumptions were corrected by logarithmic transformation of the response variable. As lower ΔCt values represent higher levels of expression, interpretation was easier by using a negative transformation of the response variable (− log (ΔCt_gene_)).

## Results

We successfully amplified the internal control genes *RSP18*, *RPL4*, *SDHA* and *PGK1,* and the three target genes HSP70, P53 and KIN (see partial gene sequences in Additional file 1). Each primer set (see Table 1) generated a single and well-defined band and had a unique dissociation curve (see Additional file 1: Figure S3). The R^2^ and amplification efficiency of all genes tested ranged from 0.991 to 1.000 and 0.92 to 101, respectively (see Table 1).

**Table 1:** Primer sequences

**Primer**	**Sequence 5′- 3′**	**GC**	**Tm**	**Size**	**Eff**	**R**^**2**^
S18-F	CAATTAAGGGTGTGGGGCGAAG*	54.5	62.1	141	99.0	1.000
S18-R	TCTTGTATTGGCGTGGATTCTGC*	47.8	60.6			
SDHA-F	TGTTTCCCACCAGGTCACACAC*	54.5	62.1	119	93.4	0.991
SDHA-R	CCAGTCGGAGCCCTTCACG*	68.4	63.1			
PGK1-F	ACAATGGAGCCAAGTCAG*	50.0	53.7	146	91.9	0.998
PGK1-R	CACGCAGTCCTTCAAGAAC*	52.6	56.7			
RPL4-F	CAGACCTTAGCAGAATCTTGAAAAGC*	42.3	61.6	171	92.0	0.998
RPL4-R	CCTGGCGAAGAATGGTGTTCC*	57.1	61.8			
HSP70-F	GTCAAGCACGGTGTTCTGTG	55.0	59.4	141	101.2	0.999
HSP70-R	CACGGCAAAGTAGAGATCATCG	50.0	60.3			
P53-F	CTCACCATCATCACACTGGA	50.0	57.3	175	94.2	0.998
P53-R	TAGGCAGTGCTCGCTTAGC	57.9	58.8			
KIN-F	TGCTGGCTTCAGAAAATCC	47.4	54.5	98	92.3	0.997
KIN-R	CTCTTGGTTCCAAAGCGTCTC	52.4	59.8			

F = forward; R = reverse; GC = GC content (percentage); Tm corresponds to the theoretical melting temperature. R^2^ corresponds to the linear correlation coefficient of the standard curve obtained by plotting the logarithm of the quantity of gene expression versus the threshold cycle (Ct). The slope of the curve was used to calculate the amplification efficiency (in%) for each pair of primers (Eff = (10^1/-slope^-1)*100). ^*^ Primers tested previously in dolphins [13].

### Stability of internal control gene expression

*Bestkeeper analysis* showed that all the selected genes were stably expressed in the epidermis of all three whale species (SD_Ct value_ ≤ 1; Table 2; Additional file 1: Figure S4) and thus can be considered as suitable control genes [37]. When considering all species together, the two most stable genes, according to their SD_Ct value_, were *RPL4*, and *RPS18* followed by *SDHA* and *PGK1* (Table 2). The four candidate genes were used for the calculation of the BestKeeper index. When pooling samples from all three species, the most suitable genes according to their coefficient of correlation were, in order, *RPS18*, *RPL4* or *PGK1* and *SDHA* (Table 2). When looking at each species separately, *RPS18* had the highest correlation coefficient in all cases, while the second best candidate gene differed amongst species, being *RPL4* for fin and sperm whales and *PGK1* for blue whales (Table 3). Sample integrity was of high quality and all intrinsic variances (InVar [±x-fold]) ranged between 0.05 and 0.97. One sample showed a higher InVar value (2.43) but was still within the acceptable range [37].

**Table 2:** Descriptive statistics of gene expression values obtained with the Bestkeeper software

	**RPS18**	**SDHA**	**PGK1**	**RPL4**
**N**	20	20	20	20
**GM**	19.23	26.91	23.44	19.10
**AM**	19.25	26.93	23.47	19.12
**Min**	17.92	25.19	21.51	17.55
**Max**	22.11	28.89	26.60	21.18
**SD**	0.84	0.84	1.00	0.70
**CV**	4.35	3.12	4.26	3.66
**Corr. coeff.**	**0.97**	0.648	0.94	0.94
**p-value**	0.001	0.002	0.001	0.001

GM (Geometric Mean), AM (Arithmetic mean), Min (Minimum), Max (Maximum), SD (Standard Deviation), CV (Coefficient of variance) of the Ct-value of the 20 samples (n) for each candidate gene. The last two rows show the coefficient of correlation (Corr.coeff.) and its p-value between the BestKeeper index and each of the candidate genes. The most reliable candidate gene is the one showing the highest correlation coefficient with the BestKeeper index (in bold).

**Table 3:** Best internal control genes for each whale species calculated with BestKeeper, geNorm and NormFinder

	**Blue whale**	**Fin whale**	**Sperm whale**
**BestKeeper**	RPS18 / PGK1	RPS18 / RPL4	RPS18 / RPL4
**geNorm**	RPS18 / RPL4	RPS18 / RPL4	RPS18 / PGK1
**NormFinder**	RPS18 / PGK1	RPS18 / RPL4	RPS18 / RPL4

**Figure 1:**
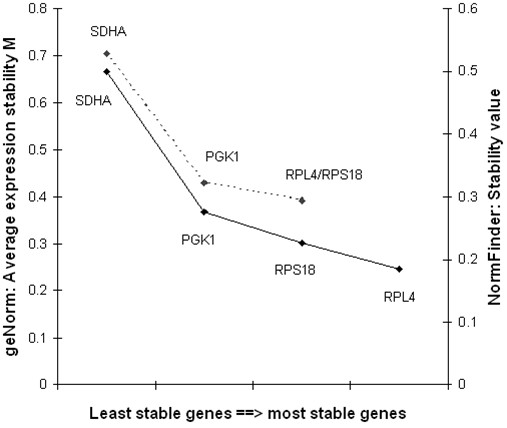
**Gene expression stability of the internal control gene candidates.** The average expression stability (M) values of the candidate genes were calculated with geNorm after stepwise exclusion of the least stable gene (left axis; M value from the least stable on the left to the most stable on the right: 0.705, 0.430 and 0.392; dotted line). The right axis corresponds to the stability values calculated with NormFinder (stability value from left, least stable, to right, most stable: 0.501, 0.275, 0.227 and 0.184; plain line).

*GeNorm analysis* showed that the expression of the four selected genes showed strong stability; the highest M value (0.98) detected (*SDHA*) being lower than the program’s default limit (M=1.5). The two most stable genes for the three species were *RPS18* and *RPL4* (Figure 1). When looking at each species separately, the best candidate genes for blue and fin whales were *RPS18* and *RPL4* whereas for sperm whales *RPS18* and *PGK1* showed higher stability (Table 3). The optimal number of control genes needed for qPCR normalization was more than four genes when pooling the three species (V_3/4_ = 0.237 > 0.15 default cut-off value), whereas when looking at each species separately, less than three genes were needed.

*NormFinder analysis* showed that when analysing all three species together, the gene with the lowest (best) stability value was *RPL4* (stability value: 0.184; Figure 1) and the most suitable gene combination was *RPS18* + *RPL4*, having a stability value of 0.184. When analysing each species separately, the best gene in all cases was *RPS18*, concurring with the results generated with the other software (Table 3).

All three software packages used concurred in selecting *RPL4* and *RPS18* as best intra- and interspecies control genes and these were used for subsequent expression analyses of target genes. The efficiencies of the target and internal control genes were within the range of the accepted 10% of each other (see Table 1), which made it possible to use the Delta Ct method [36].

### Levels of target gene expression

**Figure 2:**
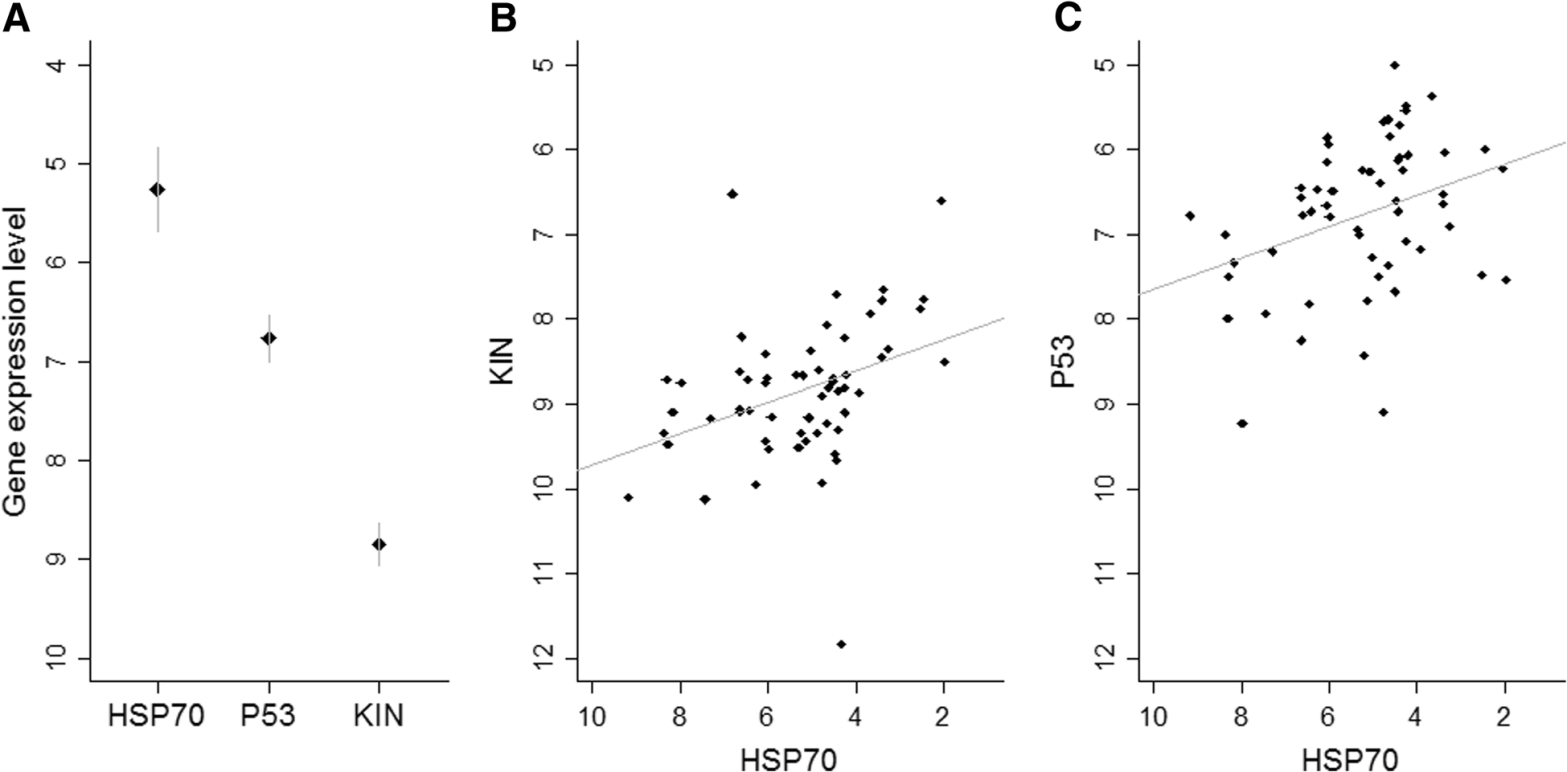
***HSP70, P53 *and *KIN *expression levels and correlations. A)** Mean levels of expression (in ΔCt, y axis inverted) of *HSP70* (n=60), *P53* (n=59) and *KIN* (n=60). Bars = ± 95% CI. **B)** Correlation between *KIN* and *HSP70* expression levels (in ΔCt). **C)** Correlation between *P53* and *HSP70* expression levels (in ΔCt). The lines show regression lines. Lower ΔCt values represent higher levels of expression.

**Figure 3:**
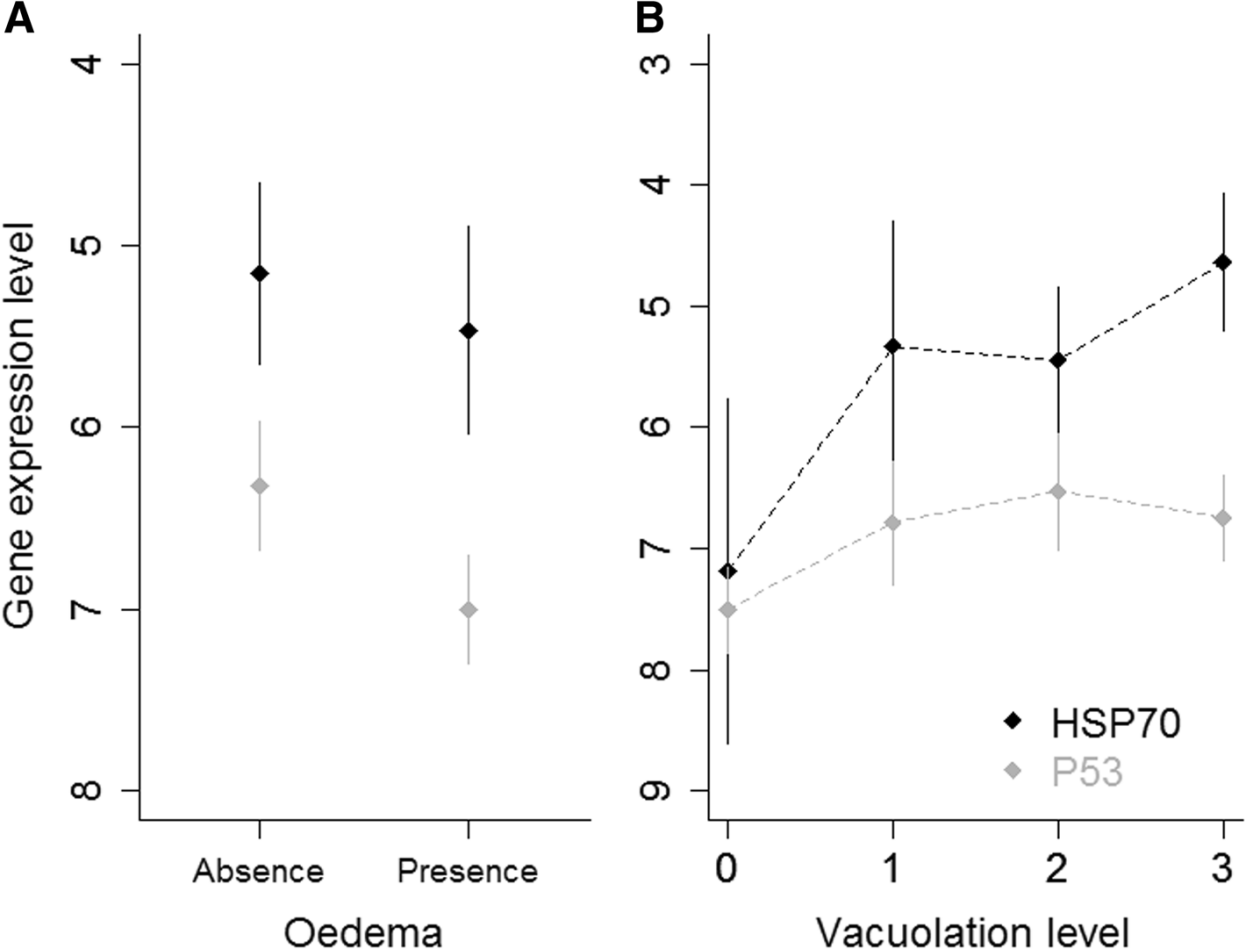
**Relation between gene expression levels and the presence of UV-induced microscopic damage. A)** Relationship between mean expression levels of *P53* and *HSP70* genes (in ΔCt, y axis is inverted) and occurrence of intracellular oedema (absence: n = 20; 1; presence: n = 37). **B)** Variation in *HSP70* and *P53* expression levels (in ΔCt, y axis inverted) for each level of cytoplasmic vacuolation (level 0 corresponding to absence and level 3 to high abundance; n = 5, 14, 20, 18 for level 0, 1, 2, 3 respectively). Bars ± 95% CI.

The gene with the highest expression level was the gene coding for the heat shock protein 70 (*HSP70*; ΔCt mean = 5.22 ± 0.21 SE, n = 60). Expression levels for *HSP70* were 1.31 times the levels observed for the tumour protein *P53* gene (ΔCt mean = 6.72 ± 0.12 SE, n = 59) and 1.69 times that of the gene coding for the KIN protein (ΔCt mean = 8.85 ± 0.11 SE, n = 60) (Figure 2A). To investigate whether gene expression levels were correlated, we fitted three mixed effects models, one for each of the target genes (Table 4). Direct relationships were observed between the expressions of *KIN* and H*SP70* and the expression of *P53* and *HSP70* (Table 4; Figure 2B and C, respectively). To investigate whether gene expression levels were related to the presence of UV-induced microscopic damage such as intracellular oedema and cytoplasmic vacuolation previously recorded (see [4] for details), we constructed three mixed effect models, one for each of the target genes (Table 5). Interestingly, the models showed that expression of *P53* and *HSP70* was lower when oedema was present (Table 5; Figure 3A). Vacuolation did not significantly predict gene expression and thus was not retained in the final models (Table 5). However, when observed graphically, there appears to be a slight trend where higher levels of gene expression tend to be observed when vacuoles are present (Figure 3B). *KIN* expression was not significantly correlated with any of the epidermal lesions included in the full model (Table 5).

**Table 4:** Likelihood ratio tests showing relationships between the expressions of the genes

***Gene***	***Expl***	***LR***	***P***
*KIN*	*P53*	0.98	0.32
	*species*	7.50	**0.02**
	*HSP70*	7.26	**<0.01**
	*plate-random*	2.55	0.11
*P53*	*KIN*	0.66	0.42
	*species*	13.72	**0.001**
	*HSP70*	15.67	**0.0001**
	*plate-random*	0.79	0.37
*HSP70*	*KIN*	3.94	**0.05**
	*P53*	8.73	**<0.005**
	*species*	23.37	**<0.0001**
	*plate-random*	6.89	**<0.01**

The Expl column lists the explanatory variables included in the full model and their corresponding likelihood ratios (LR) and p-value (p). The factors ‘plate’ (15 levels) and ‘species’ (3 levels) were fitted as explanatory variables to control for any potential effect on the variation of gene expression. Bold text indicates p ≤ 0.05. Transformed values used: - log (ΔCt_gene_).

**Table 5:** Likelihood ratio tests showing the relation between gene expression and the presence of epidermal lesions

***Gene***	***Expl***	***LRT***	***P***
P53	*oed:vac*	1.78	0.62
	*species*	1.65	0.44
	*vac*	3.85	0.28
	*oed*	7.06	**<0.01**
	*plate-random*	5.03	0.02
HSP70	*oed:vac*	1.43	0.70
	*vac*	3.03	0.39
	*oed*	5.60	**0.02**
	*species*	19.60	**0.0001**
	*plate-random*	12.70	**0.0004**
KIN	*oed:vac*	1.65	0.65
	*vac*	3.21	0.36
	*oed*	0.79	0.37
	*species*	13.63	**0.001**
	*plate-random*	2.68	0.10

The explanatory variables included in the full model were ‘oedema’ (defined as a factor with two levels: absence = intercept, presence = oed), ‘vacuolation’ (vac, defined as a factor with four levels including absence=intercept) and the interaction between the two, ‘species’ (defined as a factor with 3 levels) was fitted as an explanatory variable to control for any potential effect on the variation of gene expression. ‘Plate’ was fitted as a random factor. Bold text indicates p ≤ 0.05. In all cases, the response variable transformed was: - log (ΔCt_gene_).

## Discussion

To study the genotoxic stress pathways used by cetaceans in response to solar UV exposure, we proposed quantifying changes in the expression of key genes, namely those encoding the heat shock protein 70 (*HSP70*), an indicator of cell stress [22,25], the tumour protein 53 (*P53*), involved in most of the UV-induced gene transcription [8,29], and the KIN17 protein (*KIN*), a cell cycle control protein up-regulated by UV [32,34]. We chose qPCR to quantify mRNA transcripts because of the technique’s accuracy, sensitivity, replicability and potential to produce results rapidly [11]. However, despite its advantages, normalization of the technique, including selection of suitable internal control genes, is vital to obtain meaningful results [12]. This is particularly true when working in non-controlled field conditions, as it is extremely difficult to ensure that RNA quality will be equal for all samples.

The genes that encode the ribosomal proteins S18 (*RSP18*), ribosomal proteins L4 (*RPL4*), succinate dehydrogenase complex subunit A (*SDHA*) and phosphoglycerate kinase 1 (*PGK1*) were stably expressed and thus considered as suitable control genes. The optimal number of control genes needed for qPCR normalization was fewer than three genes when looking at each species separately and more than four genes when pooling the three study species. Although it could well be argued that our selected control gene panel is not optimal in number, due to our limited amount of sample tissue available per whale, we considered it impractical to quantify more than four control genes for studying three target genes. Financial or logistical constraints generally restrict the use of more than two internal control genes, and various studies that examine variation in target gene expression generally use two internal control genes [14,16]. In this sense, we were similarly constrained by practical issues and aimed to make the best use of our samples by incorporating genes that were relatively stable and that were known to not have a biological association with UV-related pathways as control genes. The number of house-keeping genes we selected for this study was the result of a trade-off between practical considerations and accuracy [12].

The three software packages used concurred in selecting *RSP18* and *RPL4* as best internal control genes. The combination of these two genes also presented the highest stability value. *RSP18* and *RPL4* are useful not only for studies that focus on blue, fin or sperm whales independently, but also for studies that aim to compare gene expression between the three species. It is possible that these genes are useful as control genes for other species. For instance, *RSP18* and *RPL4* showed a similar behaviour in striped dolphin skin samples and were proposed as useful reference genes [13].

In contrast to the previously published study on striped dolphins [13], the gene coding for *PGK1* was found to be a better control gene in our study than *SDHA*, which is probably due to species differences in gene expression. While *GAPDH* and *YWHAZ* were the most reliable internal control genes in striped dolphin skin [13], *SDHA* was the most reliable gene in a study that evaluated gene expression changes associated with organochlorine exposure in fibroblast cultures of striped dolphin [14]. Thus, it is clear that depending on the purpose of the study, species and target tissue to be analyzed, different genes might result more reliable as controls and thus it is essential that each study includes its own internal control gene analysis.

Although it was not possible to ascertain whether gene expression levels were constitutive or induced, *HSP70* was found to be the gene with the highest level of expression (1.3 and 1.7 times more than *P53* and *KIN,* respectively). Similar results have been observed in human melanocytes, where *HSP70* was expressed at least 2.2 fold higher than the other 11 genes involved in different pathways of DNA repair mechanisms when under UV irradiance [42]. Over-expression of *HSP70* might help initially to restore unstable or denatured proteins affected by UV stress [22]. It might also protect the cells against UV-induced damage, including apoptosis and DNA lesions [27].

Expression levels of *P53* and of *KIN* were directly related to expression of *HSP70*. It is possible that *HSP70* induces the expression of *P53* and *KIN* in whales. Alternatively, *P53* and/or *KIN* might induce expression of *HSP70*. In humans, *HSP70* closely interacts with cell-cycle arrest proteins such as P53 protein [43,44]. Although no studies on the association between *HSP70* and *KIN* have been published, it is possible that, *HSP70* may regulate KIN expression as the latter participates in the cell-cycle arrest. Chaperone proteins from the HSP70 family are known to recognize and bind mutant P53 proteins and thus regulate their accumulation and cellular localization [43,44]. Furthermore, when *P53* is mutated, its tertiary structure is modified liberating a binding domain, to which *HSP70* can bind [44]. Although HSP70-P53 complexes have been observed in carcinoma cell lines, their biological significance is still unclear [45].

Finally, the observed correlations between transcription levels of genes involved in genotoxic stress response pathways and microscopic lesions associated with excessive acute solar exposure were interesting. For instance, *P53* and *HSP70* were expressed at lower levels when intracellular oedema, a lesion generally observed in skin inflammation [4,46]. In humans and laboratory animals, it has been demonstrated that the activation of NF-kappaB, the central director of inflammation, reduces tumor suppressor activity of P53 and, thus, protects cells from P53-mediated death [46]. It is possible that occurrence of intracellular oedema has an inhibitory effect on the expression of *P53*. Likewise, the anti-inflammatory property of intracellular expression of *HSP70*[47] might explain the absence of oedema in those cells overexpressing *HSP70*. Interestingly, the presence of cytoplasmic vacuoles appeared to be directly associated with higher levels of gene expression. Although this pattern was non-significant, it is tempting to say that P53 and HSP70 are molecular tissue guardians [48] as cytoplasmic vacuolation is an alternative to the apoptotic process that aborts precancerous cells [49]. However, the cytoprotective activity of HSP70 overexpression reported in laboratory animals [50] would appear to discard this hypothesis. Exploring these associations in more depth, including protein expression assays, was beyond the scope of our study, but the observed trend suggests exciting possibilities regarding potential pathways used by cetaceans to counteract UV-induced damage.

## Conclusion

In this study we successfully standardized the quantitation of mRNA transcripts using qPCR as a proposed method to study cetacean ability to cope with UV-induced damage. Our approach for standardization included selection of the best suitable control genes as the ribosomal proteins S18 (*RSP18*), ribosomal proteins L4 (*RPL4*), selection of key genes involved in genotoxic stress pathways, which included genes encoding the heat shock protein 70 (*HSP70*), an indicator of cell stress, the tumour protein 53 (*P53*), involved in most of the UV-induced gene transcription, and the KIN17 protein (*KIN*), a cell cycle control protein up-regulated by UV. Finally, we proposed a robust mixed effect modelling approach, which allowed us to control for interplate experimental variation. In this study we also provided preliminary results that demonstrate an association between the levels of expression of target genes and sunburn microscopic lesions previously recorded in cetacean epidermis [4]. Knowing that the ozone hole over the Arctic has recently recorded its largest size [2], it is pressing to increase the number of studies looking at the effect of UV on vulnerable wildlife.

## Competing interests

The authors declare they do not have any competing interests.

## Authors’ contributions

LMML and DG collected the samples and conducted fieldwork. LMML performed all laboratory work and analysed the data. LMML and KAW designed the experiments and wrote the manuscript. KAW conceived the study. All authors discussed the study’s results, read and approved the final manuscript.

## Supplementary Material

Additional file 1 **The supplementary material is presented together as a pdf file.** The content is divided into subheadings: 1. Multiple sequence alignment (CLUSTALW) used to design the pair of primer for the three selected target genes; 2. Gene sequences included in the analyses; 3. Supplementary Figures, which include four figures: **Figure S1.** Schematic representation of the gene expression protocol; **Figure S2.** RNA integrity using the QIAxcel system; **Figure S3.** qPCR dissociation curves of the genes used in the analyses and **Figure S4.** Expression levels of the internal control gene candidates. Click here for file
