# Supplementary material for: Control and target gene selection for studies on UV-induced genotoxicity in whales
Source: BMC Res Notes. 2013 Jul 9;6:264. doi: 10.1186/1756-0500-6-264 (PMC3716943; doi:10.1186/1756-0500-6-264)
Supplement: Additional file 1 — The supplementary material is presented together as a pdf file. The content is divided into subheadings: 1. Multiple sequence alignment (CLUSTALW) used to design the pair of primer for the three selected target genes; 2. Gene sequences included in the analyses; 3. Supplementary Figures, which include four figures: Figure S1. Schematic representation of the gene expression protocol; Figure S2. RNA integrity using the QIAxcel system; Figure S3. qPCR dissociation curves of the genes used in the analyses and Figure S4. Expression levels of the internal control gene candidates. [file 1756-0500-6-264-S1.pdf]

1. Multiple sequence alignment (CLUSTALW) used to design the pair of primer for the three selected target genes.

CLUSTALW used to design the pair of primers for *P53* (underlined sequences)

Reference numbers of the NCBI sequences used:

- P53-Bos: *Bos taurus* tumor protein p53 (TP53), mRNA [NCBI Reference Sequence: NM_174201.2]
- P53-Delphinapterus: *Delphinapterus leucas* P53 (p53) mRNA, complete cds [GenBank: AF475081.1]
- P53-Sus: *Sus scrofa* tumor suppressor p53 (p53) mRNA, complete cds [GenBank: AF098067.1]
- P53-Homo: *Homo sapiens* tumor protein p53 (TP53), transcript variant 7, mRNA [NCBI Reference Sequence: NM_001126117.1]

```
P53-Bos                 TAACAGCTCCTGCATGGGGGGCATGAACCGGCGGCCCATCCTCACCATCATCACACTGGA 893
P53-Delphinapterus      TAACAGCTCCTGCATGGGAGGCATGAACCGGCGGCCCATCCTCACCATCATCACACTGGA 755
P53-Sus                 CAACAGCTCCTGCATGGGGGGCATGAACCGGCGGCCCATCCTCACCATCATCACACTGGA 754
P53-Homo                TAACAGTTCCTGCATGGGCGGCATGAACCGGAGGCCCATCCTCACCATCATCACACTGGA 655
                         ***** *********** ************ ****************************

P53-Bos                 AGACTCTTGTGGTAACCTGCTGGGACGGAACAGCTTTGAGGTGCGTGTTTGTGCCTGTCC 953
P53-Delphinapterus      AGACTCCAATGGTAATCTGCTGGGACGGAACAGCTTTGAGGTGCGTGTTTGTGCGTGTCC 815
P53-Sus                 AGACGCCAGTGGCAACTTGCTGGGACGGAACAGCTTTGAGGTGCGTGTTTGTGCCTGTCC 814
P53-Homo                AGACTCCAGTGGTAATCTACTGGGACGGAACAGCTTTGAGGTGCGTGTTTGTGCCTGTCC 715
                        **** *   *** **  * *********************************** *****

P53-Bos                 TGGGAGAGACCGCCGCACTGAGGAAGAAAATCTTCGCAAGAAGGGGCAGTCTTGCCCCGA 1013
P53-Delphinapterus      TGGGAGAGACCGCCGCACAGAGGAAGAAAATTTCCACAAGAAGGGGCAGTCTTGCCCTGA 875
P53-Sus                 TGGGAGAGACCGCCGCACAGAGGAAGAAAATTTCCTCAAGAAGGGCCAGTCTTGCCCCGA 874
P53-Homo                TGGGAGAGACCGGCGCACAGAGGAAGAGAATCTCCGCAAGAAAGGGGAGCCTCACCACGA 775
                        ************ ***** ******** *** * * ****** **  ** **  **  **

P53-Bos                 GCCACCCCCTAGGAGCACTAAGCGAGCACTGCCTACCAACACCAGCTCCTCTCCACAGCC 1073
P53-Delphinapterus      GCTGCCCACTGGGAGCGCTAAGCGAGCACTGCCTACCGGCACCAGCTCCTCTCCACCGCA 935
P53-Sus                 GCCGCCCCCTGGCAGCACTAAGCGAGCACTGCCCACCAGCACCAGCTCCTCGCCAGTGCA 934
P53-Homo                GCTGCCCCCAGGGAGCACTAAGCGAGCACTGCCCAACAACACCAGCTCCTCTCCCCAGCC 835
                        **  *** *  * *** **************** * *  ************ **   **
```

CLUSTALW used to design the pair of primers for *KIN* (underlined sequences)

Reference numbers of the NCBI sequences used:

- KIN-Pan: PREDICTED: *Pan troglodytes* KIN, antigenic determinant of recA protein homolog (mouse), transcript variant 1 (KIN), mRNA [NCBI Reference Sequence: XM_001148768.2]
- KIN-Equus: PREDICTED: *Equus caballus* KIN, antigenic determinant of recA protein homolog (mouse) (KIN), mRNA [NCBI Reference Sequence: XM_001916725.2]
- KIN-Mus: *Mus musculus* antigenic determinant of rec-A protein (Kin), mRNA [NCBI Reference Sequence: NM_025280.2]

- KIN-Homo: *Homo sapiens* cDNA FLJ58083 complete cds, highly similar to Homo sapiens KIN, antigenic determinant of recA protein homolog, mRNA [GenBank: AK301789.1]
- KIN-Bos: *Bos taurus* KIN, antigenic determinant of recA protein homolog (mouse), mRNA (cDNA clone MGC:159891 IMAGE:8492887), complete cds [GenBank: BC146082.1]

```
KIN-Pan          TCATCAGAGACAACTATTGCTGGCTTCAGAAAATCCTCAGCAGTTTATGGATTATTTTTC 257
KIN-Equus        TCATCAGAGACAACTATTGCTGGCGTCAGAAAATCCTCAGCAGTTTATGGATTATTTTTC 206
KIN-Mus          TCATCAAAGACAACTGTTGCTGGCTTCAGAAAACCCTCAGCAGTTTATGGATTATTTTTC 225
KIN-Homo         TCATCAGAGACAACTATTGCTGGCTTCAGAAAATCCTCAGCAGTTTATGGATTATTTTTC 300
KIN-Bos          TCATCAGAGACAGCTGTTGCTGGCTTCAGAAAATCCTCAGCAGTTTATGGATTATTTTTC 247
                 ****** ***** ** ******** ******** **************************

KIN-Pan          AGAGGAATTCCGAAATGACTTTCTAGAACTTCTCAGGAGACGCTTTGGCACTAAAAGGGT 317
KIN-Equus        AGAGGAATTCCGAAATGACTTCCTGGAACTTCTCAGGAGGCGCTTTGGCACTAAGAGAGT 266
KIN-Mus          AGAGGAATTCCGAAATGACTTTCTGGAACTTCTGAGGCGACGCTTTGGCACTAAAAGGGT 285
KIN-Homo         AGAGGAATTCCGAAATGACTTTCTAGAACTTCTCAGGAGACGCTTTGGCACTAAAAGGGT 360
KIN-Bos          AGAGGAATTCCGAAATGACTTCCTAGAACTTCTCAGGAGACGCTTTGGCACCAAGAGGGT 307
                 ********************* ** ******** *** * *********** ** ** **
```

HSP70 primers design

For HSP70, we designed the pair of primers directly using a sequence of *HSP70* from a right whale (see reference below).

The CLUSTALW below shows where the primers (underlined sequences) were designed on the right whale sequence. It also shows the alignment of our sequence (HSP70-Balaenoptera) with the right whale sequence and the sequence of a dolphin (see reference numbers below).

Reference numbers of the NCBI sequences used:

- HSP70-Eubalaena: est_e_glacialis2590 RightWhaleSkin *Eubalaena glacialis* cDNA, mRNA sequence [GenBank: ES556841.1]
- Tursiops: *Tursiops truncatus* Contig167074, whole genome shotgun sequence [GenBank: ABRN02157953.1]
- HSP70-Balaenoptera: *Balaenoptera musculus* Heat shock protein 70 (*HSP70*) mRNA, partial cds (sequence obtained for this study)

```
HSP70-Eubalaena      CCAAACTTGCGGCCGATCAGCCGCTTCGCGTCAAGCACGGTGTTCTGTGGGTTCAGCGCC 491
HSP70-Balaenoptera   -----------------------------GTCAAGCACGGTGTTCTGTGGGTTCAGCGCC 31
Tursiops             CCAAACTTGCGGCCGATCAGCCGCTTCGCGTCGAACACGGTGTTCTGTGGGTTCAGCGCC 4980
                                                  *** * *************************

HSP70-Eubalaena      ACCTGGTTCTTGGCCGCGTCGCCGATGAGCCGCTCGGTGTCCGTGAAGGCCACGTAGCTG 551
HSP70-Balaenoptera   ACCTGGTTCTTGGCCGCGTCGCCGATGAGCCGCTCGGTGTCCGTGAAGGCCACGTAGCTG 91
Tursiops             ACCTGGTTCTTGGCCGCGTCGCCGATGAGCCGCTCGGTGTCCGTGAAGGCCACGTAGCTG 5040
                     ************************************************************

HSP70-Eubalaena      GGGGTGGTGCGGTTGCCCTGATCGTTGGCGATGATCTCTACTTTGCCGTGCCTGAAACAC 611
HSP70-Balaenoptera   GGGGTGGTGCGGTTGCCCTGATCGTTGGCGATGATCTCTACTTTGCCGTG---------- 141
Tursiops             GGGGTGGTGCGGTTGCCCTGATCGTTGGCGATGATCTCTACTTTGCCGTGC-TGGAACAC 5099
                     **************************************************
```

2. Gene sequences included in the analyses

The following sequences correspond to the section of the genes used in the qPCR analyses. The primers are underlined.

>Seq1 [organism=*Balaenoptera physalus*] Tumour protein 53 (*P53*) mRNA, partial cds for Seq1
CTCACCATCATCACACTGGAAGACTCCAGTGGTAATCTGCTGGGACGGAACAGCT
TTGAGGTGCGTGTTTGTGCCTGTCCTGGGAGAGACCGCCGCACAGAGGAAGAAA
ATTTCCGCAAGAAGGGGCAGTCTGGCCCTGAGCCGCCTCCTGGGAGCGCTAAGCG
AGCACTGCCTA

>Seq2 [organism=*Balaenoptera musculus*] Heat shock protein 70 (*HSP70*) mRNA, partial cds for Seq2
GTCAAGCACGGTGTTCTGTGGGTTCAGCGCCACCTGGTTCTTGGCCGCGTCGCCG
ATGAGCCGCTCGGTGTCCGTGAAGGCCACGTAGCTGGGGGTGGTGCGGTTGCCCT
GATCGTTGGCGATGATCTCTACTTTGCCGTG

>Seq3 [organism=*Balaenoptera physalus*] (*KIN17*) mRNA, partial cds for Seq3
TGCTGGCTTCAGAAAATCCTCAGCAGTTTATGGATTATTTCAGAGGAATCCGAA
ATGACTTTCTAGAACTTCTCAGGAGACGCTTTGGAACCAAGAG

>Seq4 [organism=*Balaenoptera physalus*] Ribosomal protein S18 (*RPS18*) mRNA, partial cds for Seq4
CAATTAAGGGTGTGGGGCGAAGATATGCTCATGTGGTGTTGAGGAAAGCAGACA
TCGACCTCACCAAGAGGGCAGGAGAGCTCACTGAGGATGAGGTGGAACGTGTGA
TCACCATTATGCAGAATCCACGCCAATACAAGA

>Seq5 [organism=*Balaenoptera physalus*] Ribosomal protein L4 (*RPL4*) mRNA, partial cds for Seq5
CAGACCTTAGCAGAATCTTGAAAAGCCCAGAGATCCAAAGAGCCCTCCGAGCAC
CACGCAAGAAGATTCATCGCAGAGTCCTGAAGAAGAATCCACTGAAAAACCTGA
GAATCATGTTGAAGCTAAACCCATATGCAAAGACCATGCGCCGGAACACCATTCT
TCGCCAGG

>Seq6 [organism=*Balaenoptera musculus*] Succinate dehydrogenase (*SDHA*) mRNA, partial cds for Seq6
TGTTTCCCACCAGGTCACACACTGTCGCAGCCCAGGGAGGGATCAACGCCGCCCT
GGGGAACATGGAGGAGGACAACTGGAGGTGGCACTTCTACGACACCGTGAAGGG
CTCCGACTGG

>Seq7 [organism=*Balaenoptera physalus*] Phosphoglycerate kinase 1 (*PGK1*) mRNA, partial cds for Seq7
ACAATGGAGCCAAGTCAGTTGTTCTTATGAGCCACCTGGGCCGGCCTGATGGTGT
CCCCATGCCTGACAAGTACTCCTTGCAGCCAGTTGCTGTAGAACTCAAATCTCTG
CTGGGCAAGGATGTTTTGTTCTTGAAGGACTGCGTG

2. Supplementary Figures

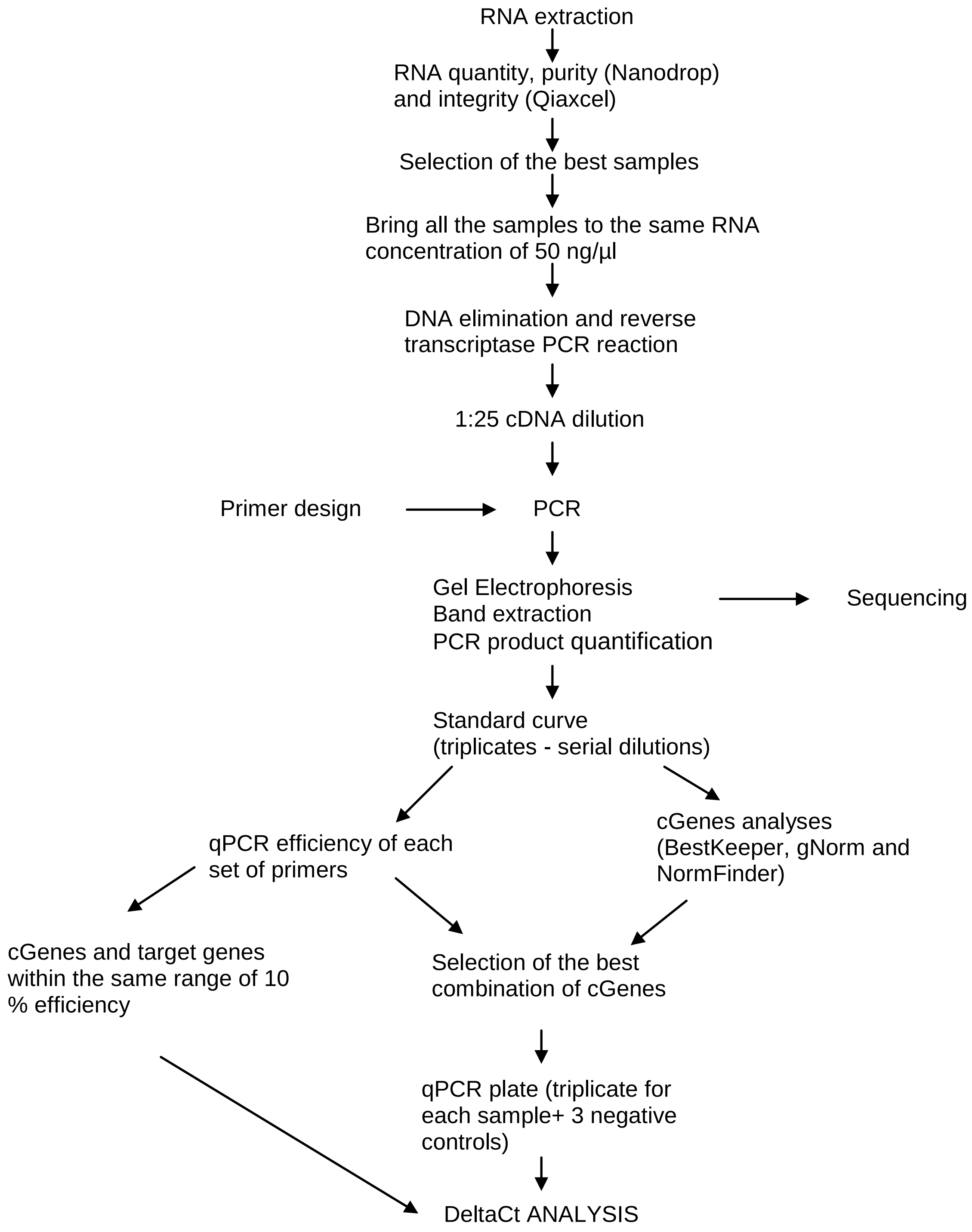

Figure S1. Schematic representation of the gene expression protocol

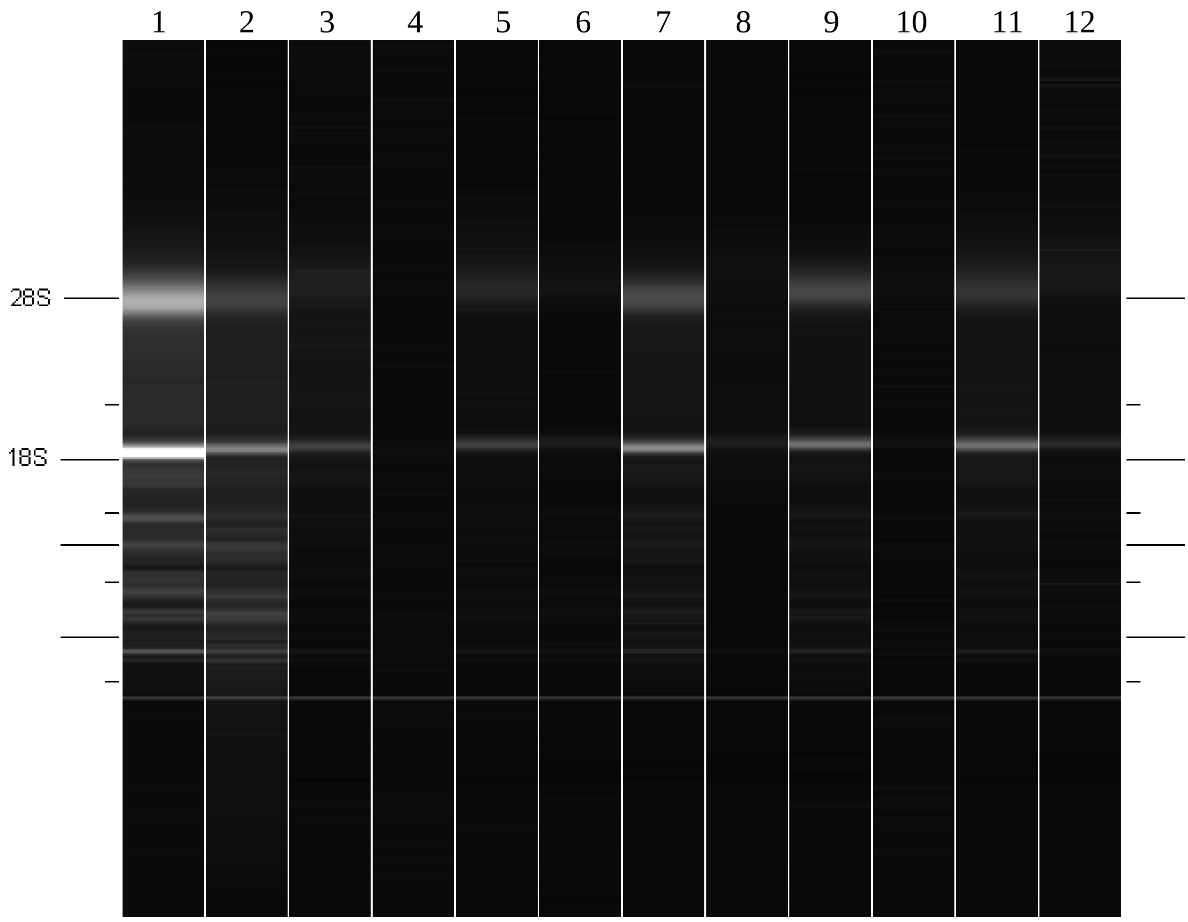

Figure S2. RNA integrity using the QIAxcel system. Intact RNA subunits 28S and 18S were observed (wells 1, 2, 3, 5, 6, 7, 9, 11 and 12) on an automated capillarity-electrophoresis system, the QIAxcel system (Qiagen,UK).

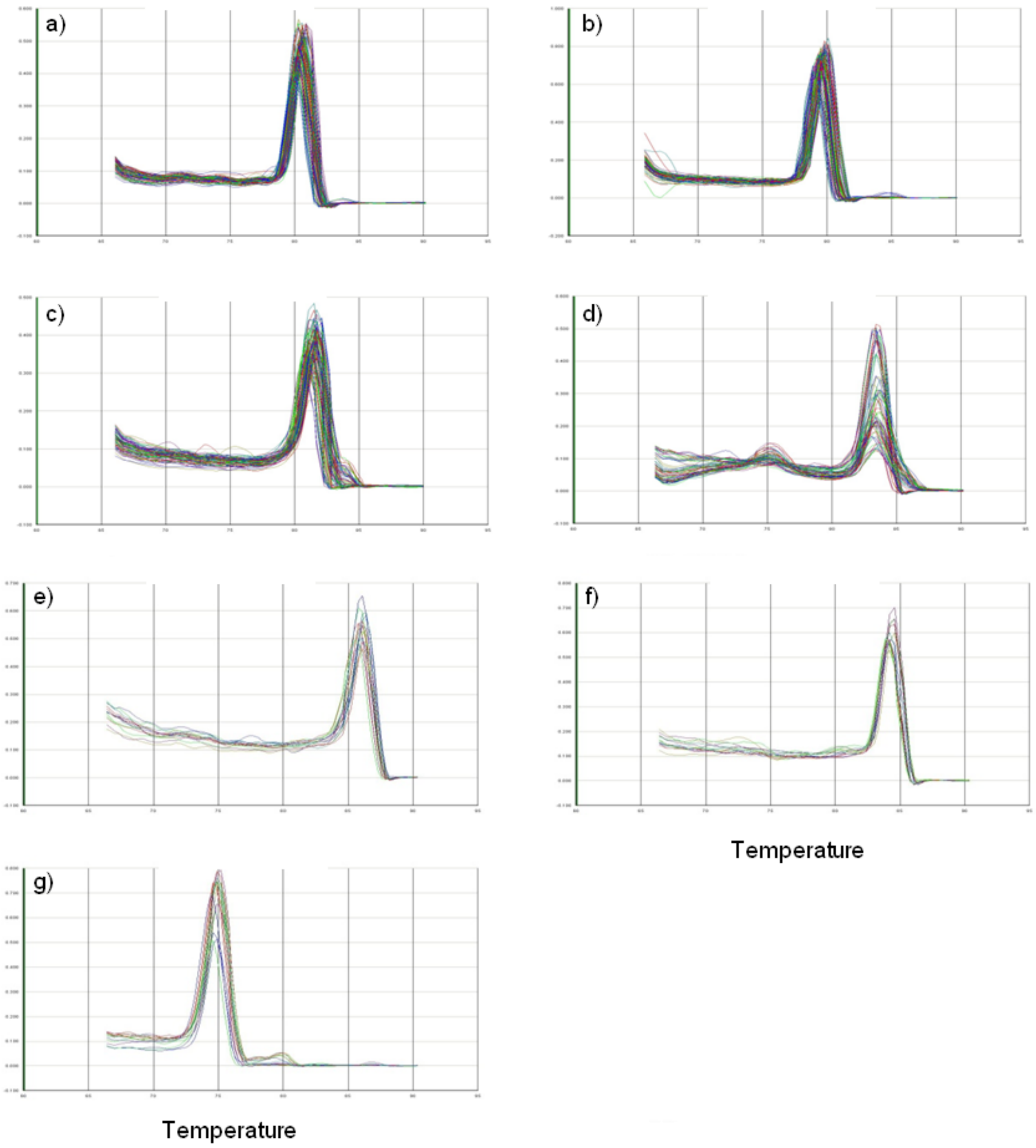

Figure S3. qPCR dissociation curves of the genes used in the analyses. Melting curve analyses images obtained with the 7300 Real-Time PCR System (Applied Biosystems) of the genes a) *RPS18*, b) *RPL4*, c) *PGK1*, d) *SDHA*, e) *HSP70*, f) *P53* and g) *KIN* showing unique product amplification. d) mild primer-dimer formation (Tm=75ºC) due to the lower level of expression of SDHA compared to the other genes.

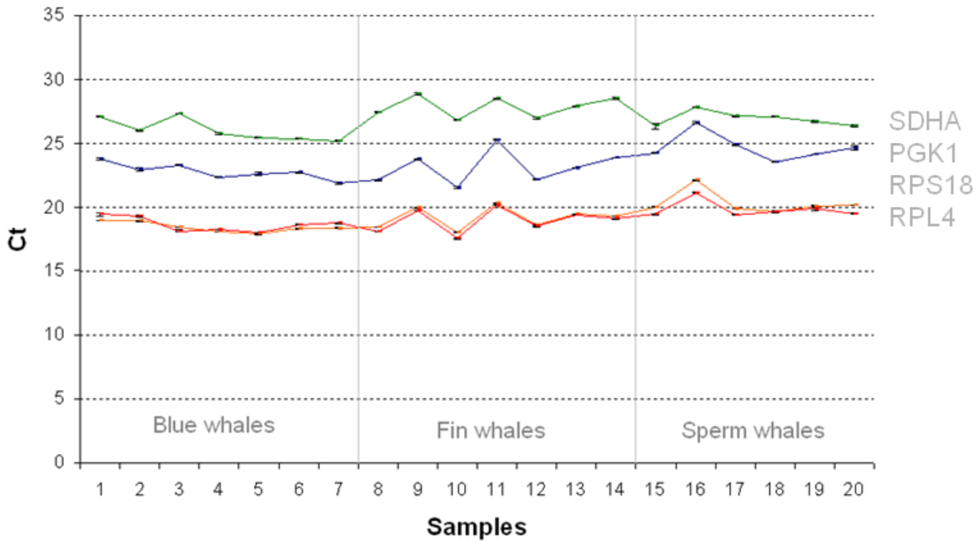

Figure S4. Expression levels of the internal control gene candidates. Mean Ct-values (left axis) for 20 skin samples of blue, fin and sperm whales (n = 7, 7 and 6, respectively). RPS18 = 19.25 ± 0.23 SE, RPL4 = 19.12 ± 0.19 SE, PGK1 = 23.47 ±0.28 SE, SDHA = 26.93 ±0.24 SE. Bars represent ± SE.
